# A Recurrent Deep Network for Gait Phase Identification from EMG Signals During Exoskeleton-Assisted Walking

**DOI:** 10.3390/s24206666

**Published:** 2024-10-16

**Authors:** Bruna Maria Vittoria Guerra, Micaela Schmid, Stefania Sozzi, Serena Pizzocaro, Alessandro Marco De Nunzio, Stefano Ramat

**Affiliations:** 1 Laboratory of Bioengineering, Department of Electrical, Computer and Biomedical Engineering, University of Pavia, 27100 Pavia, Italy; brunamariavittoria.guerra@unipv.it (B.M.V.G.); micaela.schmid@unipv.it (M.S.); stefania.sozzi@unipv.it (S.S.); spizzocaro@lunex.lu (S.P.); 2 Department of Research and Development, LUNEX International University of Health, Exercise and Sports, Avenue du Parc des Sports, 50, 4671 Differdange, Luxembourg; adenunzio@lunex.lu; 3 Luxembourg Health & Sport Sciences Research Institute ASBL, Avenue du Parc des Sports, 50, 4671 Differdange, Luxembourg

**Keywords:** gait analysis, deep learning, EMG signals, exoskeleton

## Abstract

Lower limb exoskeletons represent a relevant tool for rehabilitating gait in patients with lower limb movement disorders. Partial assistance exoskeletons adaptively provide the joint torque needed, on top of that produced by the patient, for a correct and stable gait, helping the patient to recover an autonomous gait. Thus, the device needs to identify the different phases of the gait cycle to produce precisely timed commands that drive its joint motors appropriately. In this study, EMG signals have been used for gait phase detection considering that EMG activations lead limb kinematics by at least 120 ms. We propose a deep learning model based on bidirectional LSTM to identify stance and swing gait phases from EMG data. We built a dataset of EMG signals recorded at 1500 Hz from four muscles from the dominant leg in a population of 26 healthy subjects walking overground (WO) and walking on a treadmill (WT) using a lower limb exoskeleton. The data were labeled with the corresponding stance or swing gait phase based on limb kinematics provided by inertial motion sensors. The model was studied in three different scenarios, and we explored its generalization abilities and evaluated its applicability to the online processing of EMG data. The training was always conducted on 500-sample sequences from WO recordings of 23 subjects. Testing always involved WO and WT sequences from the remaining three subjects. First, the model was trained and tested on 500 Hz EMG data, obtaining an overall accuracy on the WO and WT test datasets of 92.43% and 91.16%, respectively. The simulation of online operation required 127 ms to preprocess and classify one sequence. Second, the trained model was evaluated against a test set built on 1500 Hz EMG data. The accuracies were lower, yet the processing times were 11 ms faster. Third, we partially retrained the model on a subset of the 1500 Hz training dataset, achieving 87.17% and 89.64% accuracy on the 1500 Hz WO and WT test sets, respectively. Overall, the proposed deep learning model appears to be a valuable candidate for entering the control pipeline of a lower limb rehabilitation exoskeleton in terms of both the achieved accuracy and processing times.

## 1. Introduction

Walking is a cyclic and complex task involving multiple joints’ coordinated movement, primarily of the lower extremities [1,2]. The execution of this task relies on repeating a basic sequence of movements, called the gait cycle, which can be identified as those intervening between two nominally identical events in the process. Usually, the first event corresponds to the instant where one foot strikes the ground with the heel (heel strike event), while the second occurs when the same foot strikes the ground again [3,4]. During the gait cycle, each limb alternates one phase in which the foot is in contact with the ground, the stance phase, with one in which the foot is raised off the ground and moving forward to prepare for the next step (the swing phase).

Fluid and continuous joint motion is guaranteed by muscle activity, which represents the motor of gait [5]. Briefly, at the initial contact, the gluteus maximus and the biceps femoris (BF) are active, together with the tibialis anterior (TA). During the stance phase, several muscles work to control the ankle, the knee, and the hip joint in order to maintain balance while allowing forward progression. During early stance, the gluteus maximus, the vasti (vastus intermedius, lateralis (VL)—and medialis), and the dorsiflexors are active, whereas, during late stance, they are replaced by the gluteus medius, the soleus (SOL), and the gastrocnemius [6,7,8,9]. At the end of the stance phase, the TA prepares the initial swing phase. This latter phase involves three main muscle groups: the hip flexor muscles (adductor longus, sartorius, iliacus, and gracilis muscles), the biceps femoris (BF), the TA, and the extensor digitorum longus [6]. The action of the TA continues during the mid-swing phase to support and to maintain the ankle position. During the last part of the swing phase, three main muscles are involved: the hamstring, the rectus femoris, and the TA [1,4,10].

For gait assistance and rehabilitation, partial assistance exoskeletons are a useful tool for patients with lower limb motor disorders of various origins, such as post-stroke hemiplegia, neurodegenerative diseases such as Parkinson’s and multiple sclerosis, aging, and trauma. A partial assistance exoskeleton assists the user’s gait by interacting with the user and contributing to the intended movement [11].

This requires a synergy between the user’s intent and the device’s control, so that an appropriate exoskeleton torque or motion may be defined accordingly [12,13]. Commonly, the control of these devices involves two layers: the detection/synchronization layer aimed at estimating gait phases, which is needed as input for the second layer, that of action, which computes the motor command, and this can either be kinematic (angle or speed) or kinetic (torque or force) [13]. The output of the first layer is an estimate based on a few gait events: the heel strike and the toe mid-stanceoff, detected with a foot switch placed at the heel or the forefoot. Alternatively, these events can be recognized by analyzing the signals acquired with inertial measurement units (IMU) positioned on the lower limbs or by analyzing the muscle activity recorded by surface electrodes placed on the lower limb muscles (EMG) [14,15,16,17,18]. The estimator, which is mainly threshold-based, identifies the gait events by either processing the raw sensor output (e.g., that of the foot switch) or derived variables (e.g., joint angles) [19,20,21,22,23]. In recent years, machine learning (ML) algorithms and artificial intelligence (AI) applications have been explored as alternatives to the conventional gait analysis methods [24,25,26,27,28].

ML algorithms can map non-linear input–output relationships such as the one governing human gait [21,29,30,31,32,33,34]. When compared to conventional and heuristic methods for analyzing gait, ML implementations typically attain higher accuracy predictions, depending on the nature of the input data [35,36]. For instance, Jung et al. implemented a multilayer perceptron (MLP) neural network using IMU data to detect stance and swing phases, achieving an accuracy of approximately 97%. [36]. Ma et al. applied an MLP neural network to detect the four gait phases—heel strike, mid stance, toe off, and swing—using kinematic data computed from IMU sensors and pressure data, attaining average classification rates around 83% [37]. A similar type of data was used by Wang et al. to identify the same four gait phases by using a deep memory convolutional neural network, and they reported a model accuracy of 97.1% [25].

Hua et al. utilized an adaptive neural-fuzzy inference system (ANFIS) to detect stance and swing phases with kinematic and pressure data, enhancing the identification accuracy from 96.2% to 99.7% [38]. Zhen et al. implemented a long short-term memory network (LSTM) to detect stance and swing phases using IMU acceleration signals, achieving around 92% accuracy [39]. EMG data were also used as input to AI models for gait phase identification. Despite this type of data being more difficult to manage due to the variability of the signal amplitude, it is particularly suitable for the real-time estimation of joint position because it precedes the kinematic movement data by at least 120 milliseconds [40,41]. Hence, the use of these signals might mitigate the delay issue commonly encountered in online exoskeleton control based on kinematic data [42,43,44,45]. For instance, six different ML models, including random forest and convolutional neural networks, were implemented by Nazzari et al. for swing and stance classification using four EMG signals [46]. The average accuracy of the convolutional neural network model performed with filtered and down-sampled EMG data was 79%, just 4% higher than the best-performing ML model fed with four EMG-extracted features (standard deviation, mean absolute value, zero crossing, and mean absolute deviation). Similarly, Nazmi et al. proposed an MLP neural network with two EMG signals as input data in order to identify stance and swing gait phases. The achieved accuracy was 87.4% [47]. Several studies aiming to define an accurate predictor for gait phase classification have proposed an LSTM model. Different EMG signal features were used, generally obtaining a higher prediction accuracy than previous approaches (97.89%, 95%, 93.15%) [48,49,50].

Based on the encouraging results from such LSTM-based models, here, we propose a bidirectional LSTM architecture working on EMG signals from four muscles in the dominant leg to identify the two principal gait phases (stance and swing) with a good accuracy and roughly in real-time. The idea is to define a supervised model that is suitable to be considered for use in a partial assistance exoskeleton controller conceived for rehabilitation purposes. Specifically, our study mainly focuses on the real-time characteristic of the model prediction and on its ability to generalize over different types of walking (walking overground with an exoskeleton (WO) and on a treadmill with an exoskeleton (WT)). Numerous studies, comparing treadmill and overground walking, suggest that when individuals walk on a treadmill, they modify their muscle activation patterns and consequently their joint moments and powers while maintaining relatively constant limb kinematics and spatiotemporal gait parameters [14,51,52,53]. However, this is still an open debate despite the treadmill, compared with overground gait, being a more widely used exercise in rehabilitation programs for its numerous advantages. Consequently, to explore its generalization abilities, the model was trained on WO EMG data and was then tested separately on WO and WT EMG data [51]. At the same time, using EMG data instead of IMU or pressure data allowed us to address the constraint of real-time prediction while taking advantage of the physiological anticipation of muscle activity with respect to the kinematics of the limb.

## 2. Material and Methods

### 2.1. Subjects

Twenty-six young and healthy subjects were enrolled, following the inclusion and exclusion criteria schematically reported in Table 1, from the student population of LUNEX, Differdange, Luxembourg. Their mean (±standard deviation (SD)) age, height, and weight were 23 ± 3 years, 173 ± 10 cm, and 69 ± 14 kg. All the subjects signed the informed consent form. This study received approval from the Comité National d’Ethique de Recherche (CNER-Luxembourg National Research Ethics Committee) with protocol number 202103/02, in accordance with the Helsinki Declaration.

### 2.2. Study Design

A two-session cross-sectional study was conducted to measure lower limb muscle activity during gait with an exoskeleton (ExoAtlet, Esch-sur-Alzette, Luxembourg). Subjects were asked to perform walking tasks in two different conditions, acquired in random order, in two distinct acquisition sessions: WO and WT. 

Before investigating the performance of the model on EMG data acquired during gait in neurological patients using the exoskeleton (topic for future studies), we studied the performance of the proposed neural network considering EMG data acquired on a group of healthy subjects walking with the exoskeleton. Previous studies demonstrated that exoskeleton wearing alters the natural gait patterns, including timing, posture, and muscle synergies [14,51,52,53]. Moreover, the mechanical structure of the exoskeleton could interfere with the EMG electrodes, generating artefacts on the EMG signals. Therefore, using EMG data of healthy subjects acquired in such an ecological setting allowed the network learning process to rely on real cases and not on stereotyped and less noisy normal walking.

The anthropometric data of each subject were measured, such as height, weight, hip-to-hip distance, greater trochanter to lateral epicondyle distance (bilaterally), and lateral epicondyle to foot sole distance (bilaterally). These measures were used to personalize the length of the exoskeleton segments to the participant’s body. The dominant leg (DL) was assessed by asking participants to kick a ball and hit a target without providing additional instructions on how to perform the task [55]. Before the first gait-acquisition session, the experimenter equipped the DL of the subject with four EMG electrode pairs (Ceracarta, Forlì, Italia) and then asked them to perform a maximal voluntary isometric contraction (MVIC) in turns with TA, VL, SOL, and BF. Each participant performed three 5 s MVICs in isometric conditions with a 60 s break for each contraction [56]. The order of muscular contractions for each subject was established through a free randomization software app (Random Generator, 2.1.9 version, Apps n Blue, Jordan). The details concerning the MVIC test are described in the corresponding subparagraph at the end of this section.

At the beginning of the walking session, participants were dressed with IMU probes and EMG electrodes and were asked to complete a 10 min warm-up walk on a treadmill set at a fast walking pace (from 4 to 6 km/h). After completing the warm-up, the experimenter securely strapped the participant to the powered lower limb exoskeleton, while in sitting position, to start a 10 min familiarization session. During this period, subjects walked overground sustained by a driver always following them and holding the handles of the exoskeleton. Crutches were given to the subjects for additional safety. The familiarization session allowed the participants to get used to the sensations of limited motion induced by the exoskeleton, learn how to shift their body weight from one foot to another, and follow the exoskeleton movements without resisting them [57].

**Figure 1:**
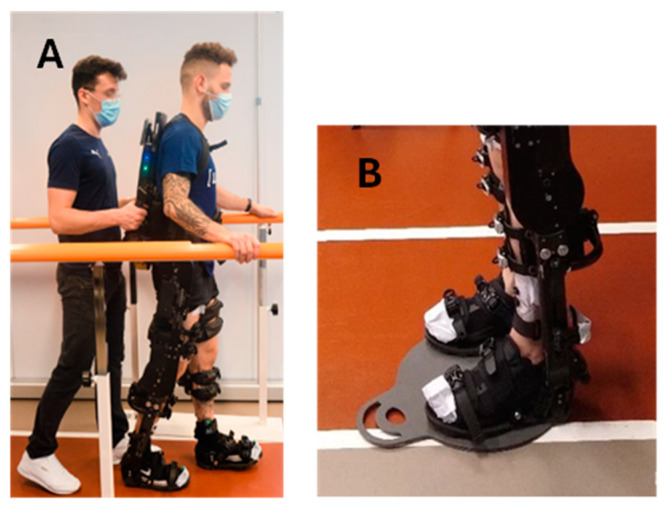
(**A**): WO between the safety bars. The experimenter follows the subject, driving the exoskeleton through the control handles. (**B**): platform for patient rotation used to invert the walking direction while the subject maintains standing position.

As soon as the familiarization session was concluded, the participants performed the walking session overground or on the treadmill. During the WO task, lasting 60 min, the experimenter followed the participant closely while holding the control handles on the back of the exoskeleton (Figure 1A). These allowed the experimenter to start and stop the exoskeleton when needed. The WO task involved walking along a straight path measuring 10 m in length and 1 m in width, maintaining an average speed of 1.3 km/h. To ensure safety, two rehabilitation parallel bars were positioned on each side of the walking path (Figure 1A). Upon reaching the end of the linear walking path, the exoskeleton was stopped via the control handles and the subject was passively rotated to invert the walking direction. This maneuver occurred with the subject unmoving in a standing position on a rotational platform for patient mobilization (Disco Duo, Chinesport SPA, Udine, Italy) (Figure 1B).

During the WT task, also lasting 60 min, subjects walked on a rehabilitation treadmill (Gait Trainer 3, BIODEX, Shirley, New York, NY, USA) fitted with safety lateral bars. The treadmill was set to an average constant speed of 1.3 km/h, the same used during the WO. 

Muscular activity of the TA, VL, SOL, and BF of DL was recorded at 1500 Hz using a wireless sEMG system (MyoMuscle, NORAXON, Scottsdale, AZ, USA) during MVIC test and WO and WT tasks. Additionally, hip and knee flexion motion data were recorded with the inertial measurement unit (IMU) sensors placed on the subject’s sacrum, DL thigh (frontal and distal half), and shank (along the tibia), sampling at 100 Hz (MyoMotion, NORAXON, Scottsdale, AZ, USA). The two devices were synchronized via software (MyoResearch, NORAXON, Scottsdale, AZ, USA). During the gait tasks, across the entire trial duration, muscular activity and IMU data were collected for three minutes every eight minutes, resulting in seven windows of three-minute acquisitions. 

Each experimental recording session (WO or WT) for each subject lasted between one and a half and two hours, with a minimum interval of twenty-one hours between the two sessions.

#### Maximal Voluntary Isometric Contraction Tasks

The MVIC test set-up guaranteed that the tested muscle remained in isometric condition while the subjects were pushing or pulling against an unmovable resistance (wall bars). The maximal developed force was acquired with an electronic dynamometer (K-Link, K-Invent, France) equipped with a dedicated app.

Ankle dorsiflexion setup (MVIC for TA)

**Figure 2:**
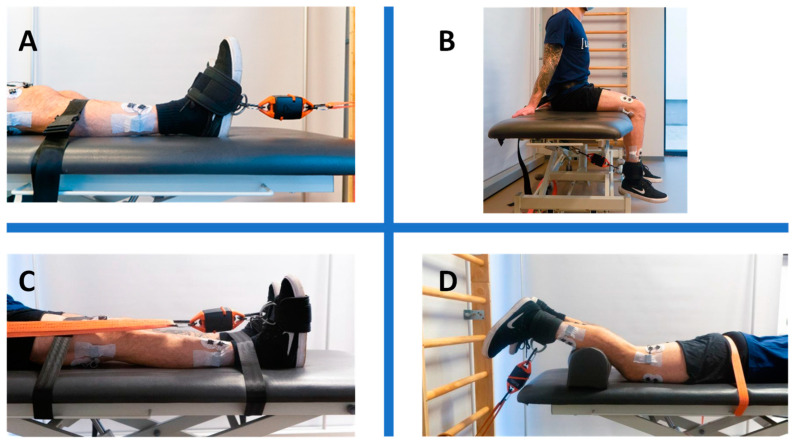
(**A**): ankle dorsiflexion setup, MVIC assessment for TA; (**B**): knee extension setup, MVIC assessment for VL; (**C**): ankle plantarflexion setup, MVIC assessment for SOL; (**D**): knee flexion setup, MVIC assessment for BF.

As shown in Figure 2A, the subject sat on the medical plinth, facing the wall bars. Three belts were strapped around the subject’s waist and above and beneath the knees to avoid participant frontal displacement and prevent knee flexion. The cuff was tightened around the foot, touching the ankle fold. The dynamometer linked the cuff to another belt attached to the wall bars.

Knee extension (MVIC for VL)

The participant assumed a seated position on the medical examination table, aligning the popliteal fossa with the lateral edge of the plinth while maintaining the knee at a precise 90° angle, as depicted in Figure 2B. A securing belt was affixed around the subject’s waist and tightly fastened to the metal frame of the plinth. Simultaneously, a cuff was securely strapped around the participant’s ankle. The dynamometer was intricately connected to both the cuff and the plinth metal frame by means of a double snap-hooked band. The participant was provided with instructions to exert maximal effort in extending the knee during each of the three 5 s bouts.

Ankle plantarflexion (MVIC for SOL)

The subject sat on the medical plinth, resting their back on a pillow protecting the bars for more comfort. As shown in Figure 2C, two belts were tightened around the subject’s thighs and ankles. The cuff was wrapped around the foot at the metatarsal level. The dynamometer linked the cuff to a belt attached to the wall bars. The subject was instructed to keep their arms crossed to their chest while exerting maximal effort in pushing on the cuff during each of the three 5 s bouts.

Knee flexion (MVIC for BF)

The participant assumed a prone position on the medical examination table, with the malleoli precisely aligned to its edge. Maintaining a 30° angle at the knees was facilitated by the placement of a pillow under the tibias, as visually represented in Figure 2D. A belt was utilized to stabilize the subject’s pelvis, preventing anterior pelvic tilt. The cuff was strategically positioned around the ankle, and an additional belt connected the dynamometer to the wall bar, ensuring a tension line nearly perpendicular to the tibia. The task assigned to the participant was to actively flex the knee, bringing the heel towards the buttocks while exerting maximal effort during each of the three 5 s intervals.

### 2.3. Exoskelteon Description

The powered lower limb exoskeleton used in this study was the ExoAtlet II from ExoAtlet Global SA, Luxembourg [58]. The ExoAtlet II (Figure 1A) is a CE-marked device designed to assist lower limb motor functions of people with walking disabilities of neurological or musculoskeletal nature in a rehabilitation context. The ExoAtlet II has a metallic structure that surrounds the user’s lower limbs and trunk. Four electric motors and mechanical actuators move the hip and the knee bilaterally, providing two degrees of freedom each. The ExoAtlet II ankle joint operates in a passive mode. To ensure stability, the user is secured to the metallic structure using a series of straps and belts. The segments are adjustable: shank and thigh length and pelvis width can be tailored to the physical characteristics of the subject’s body. The total weight of ExoAtlet II is around 33 kg.

The ExoAtlet II is controlled by a detachable tablet, which allows for the selection of different operating modes, including standing up, sitting down, stepping in place, walking, and climbing upstairs and downstairs. Depending on the selected operating mode, different motion parameters can be adjusted to modify the duration of the movement or the steps’ characteristics (length, height, etc.). The parameters set for this study are listed in Table 2. The indicated set of parameters was chosen to keep a natural gait pattern for all the participants, following the methods in [57]. Independently of the operating mode, the ExoAtlet II starts moving the left leg. The initiation of every modality is signaled with three high-pitched alerting beeps. For further details about the ExoAtlet II, including its set-up and applications, please refer to Pais-Vieira et al. [57] and Baptista et al. [59].

### 2.4. Data Processing

MVIC data collected via the electronic dynamometer were resampled at 1500 Hz and analyzed to compute the average expressed force (AEF). AEF was defined as the average of the force values over a 1 s window. For each of the three maximal contractions, the 4th second of contraction was used to avoid transients in force assessment. For each acquired muscle, the maximum of the three AEF values was considered. The sEMG data corresponding to these trials were used to compute the reference values for the sEMG data normalization [60]. sEMG data of both MVIC and walking trials were pre-processed as follows: the mean value was removed from the signals, and a bandpass Butterworth filter of the 4th order with a bandwidth of 30–400 Hz was applied forward and backwards, to avoid delays [61]. The envelope of the signals was then extracted through a combination of rectification and a 5 Hz low-pass Butterworth filter of the 4th order, applied bidirectionally [62,63,64]. The sEMG signals of each muscle were then normalized with the corresponding sEMG MVIC value, which was computed as the average amplitude of the sEMG envelope over a 250-millisecond window centered around the envelope peak.

After normalization, each sEMG signal was down-sampled to a frequency of 500 Hz by taking every 3rd sample of data. This was done to reduce the length of the input sequences and, hence, the amount of data to process for each sequence while also simplifying the task of keeping salient information throughout the input sequences for the LSTM model.

**Figure 3:**
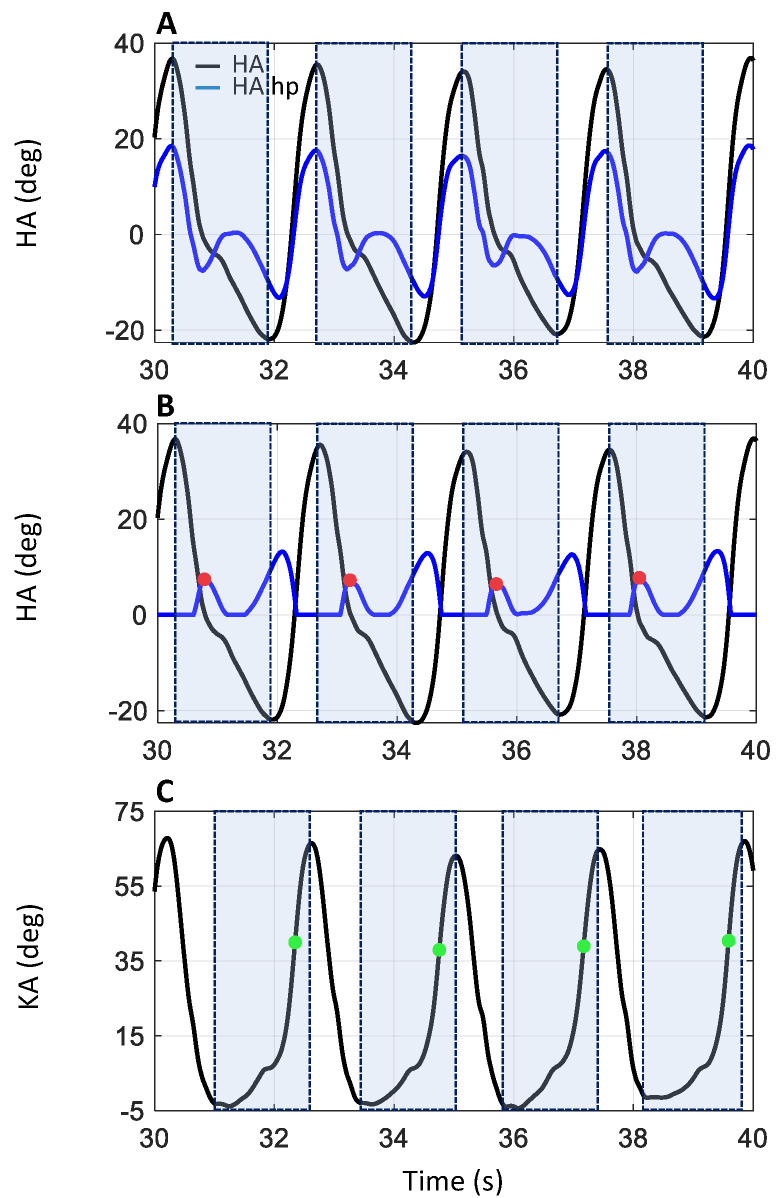
HS and TO identification. (**A**) shows the hip angle (HA) calculated between the sacrum and thigh (black trace) and the HA after being high pass-filtered (HA hp, blue trace). Positive values signify hip flexion, while negative values indicate hip extension. The positive part of the filtered HA signal was set to zero, and then the signal obtained was rectified (blue trace, (**B**)). On this trace, the local maxima within each time window defined between each maximum and the following minimum of the HA signal (pale-colored rectangles in (**A**,**B**)) were identified (red dots in (**B**)). The instants corresponding to local maxima thus identified were the instants in which the HS event occurred. In (**C**), the black trace corresponded to the knee angle (KA). Positive values signify knee flexion, while negative values indicate knee extension. In a time window between each minimum and successive maximum of the KA signal (pale-colored rectangles in (**C**)), 60% of the KA maximum was searched (green dots in (**C**)). TO occurred at 60% of the KA maximum. Only a 10 s portion of the signals is presented in the figure for clarity.

**Figure 4:**
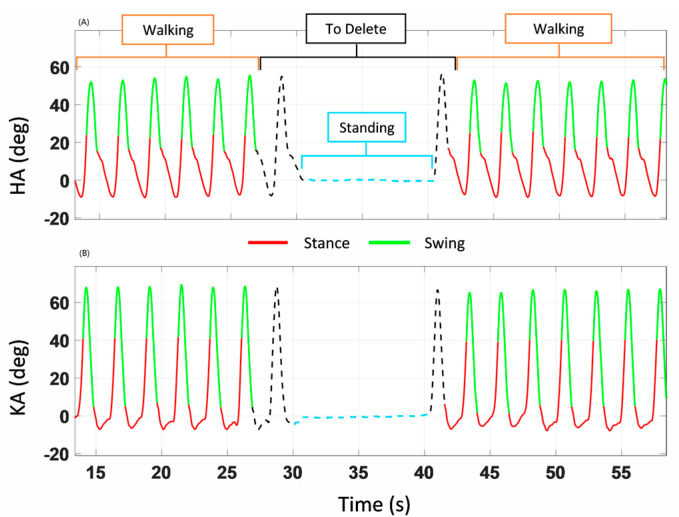
(**A**,**B**) illustrates labelled HA and KA data referring to WO condition. The light blue line labelled “Standing” corresponds to the quiet standing phase identified by the hidden Markov model. The data belonging to the stance phase are displayed in red, whereas those of the swing phase are in green. The dashed black line corresponds to the data excluded from the muscle labelling procedure, then discarded.

IMU data were processed (MyoResearch, NORAXON, Scottsdale, AZ, USA) to compute the hip angle (HA: between sacrum and dominant leg’s thigh) and the knee angle (KA: between thigh and shank), shown in Figure 3A,C, respectively. HA and KA data were analyzed to discriminate the heel strike (HS: onset of the stance phase) and the toe off event (TO: end of the stance phase) of each dominant leg stance phase. The data corresponding to the quiet standing phases (light blue dash line in Figure 4), when the subject was inverting the walking direction, were discarded. A hidden Markov model (HMM) algorithm was applied to the HA data to distinguish between the data belonging to the walking (the orange portion, labeled “Walking” in Figure 4) and the quiet standing phases (the light blue portion, labeled “Standing” in Figure 4). Specifically, an HMM with categorical emissions consisting of two types of variables, i.e., hidden states (walking and standing phase) and a discrete set of emitted symbols describing the HA, was used. The idea was to convert the original HA signal into a discrete series of symbols which represent the observable variables. The classification results of the HMM procedure are presented in Figure 4A,B, where the portions of the HA and KA traces corresponding to the quiet standing phase are highlighted in a light blue dash line and by the light blue portion labelled “Standing”. The procedure was not applied to the WT data, as the quiet standing phases were missing from these.

The HS events were identified for both WO and WT tasks by analyzing HA data. In accordance with Sylos-Labini et al. [53], for each gait cycle, the first local minimum following the maximum of the HA was taken as the HS event. To automatically detect these events, the HA data were divided into a sequence of time windows, each one defined between the local maximum of greater value and the local minimum of lower value (see the pale-colored rectangles in Figure 3A,B. Then, the HA data, shown as the black trace in Figure 3A, were processed using a high pass 2nd-order Butterworth filter with a cutoff frequency of 0.5 Hz (HA hp, blue trace in Figure 3A). The positive part of the signal was set to zero, and the resulting signal was rectified (blue trace in Figure 3B). The HS events were identified, inside each previously defined time window, as the first local maximum of the filtered and rectified HA, corresponding to the beginning of the flexion in the HA signal (see red dots in Figure 3B).

For both WO and WT tasks, the TO events were identified by analyzing KA data (Figure 3C). During the terminal stance of the gait cycle, the knee starts to flex, reaching a value of about 39° at the end of the stance phase, when the foot leaves the ground (TO event), roughly at 60% of the gait cycle. During the mid-swing phases, at about 75% of the gait cycle, the knee angle reaches a maximum peak of approximately 65° [1,4,63,65]. Based on these notions, the TO event can be estimated to occur around 60% of KA peak flexion. Therefore, for the TO detection, the data were divided into time windows defined as the interval between the first local minimum preceding peak knee flexion and peak knee flexion itself (see the pale-colored rectangles in Figure 3C). Then, the time instant corresponding to 60% of the value of the local maximum peak was sought for within the window and labeled as the TO event (green dots in Figure 3C).

### 2.5. Data Labelling and Dataset Costruction

Two distinct sEMG datasets were created: one for WO data, used for training and testing the deep learning model, and another for WT data, which was exclusively used in the model testing procedure. For the WO dataset, only sEMG data corresponding to walking activities were considered, and data from the quiet standing phase on the rotation platform were excluded. To further refine the dataset and minimize the impact of transient velocity changes, such as deceleration when approaching the rotation platform, the strides immediately before and after the quiet standing phase were removed. This approach ensured that only steps corresponding to steady-state walking were included.

First experiment

To efficiently train the deep learning model, we decided to down-sample the EMG signals, reducing the sampling rate to 500 Hz. This choice was supported by the fact that the bandwidth of the EMG signal frequency is between 0 and 500 Hz, with the dominant frequencies being between 50 and 150 Hz [61,66,67,68,69]. Therefore, with a sampling frequency of 500 Hz, the signal information content is preserved, while the shortening of the input sequences is expected to simplify the learning task of the network. 

The WO dataset was composed of a number of samples covering a total of 406,948 s (274,648 s of stance and 132,300 s of swing). The dataset was built using the cross-subject technique: 23 subjects made up the training dataset (20% of their data was set aside for validation purposes), and the remaining 3 subjects made up the test dataset. Furthermore, due to the consistent class imbalance observed in the training data (WO), a data augmentation technique based on replicating the sequences containing a prevalence >70% of minority class samples was applied. Inevitably, such a procedure also increases the number of samples representing the stance phase, although to a lesser extent. After data augmentation, the training dataset presented 300,772 s belonging to the stance phase class and 214,429 s to the swing phase class, for a total of 515,201 s of data. Thus, as the input of the deep learning model consisted of sequences of 500 samples each (1 s), and those used for training the model were built with 50% overlap, the training sequences comprised 772,801 s. The test dataset sequences corresponded instead to 4398 s and were not further processed.

The WT test dataset referred to the WT data of the same three subjects chosen for the WO test set, maintaining the experiment’s cross-subject design. It consisted of data covering 6954 s, with 4726 s belonging to the stance phase class and 2228 s to the swing phase class.

**Figure 5:**
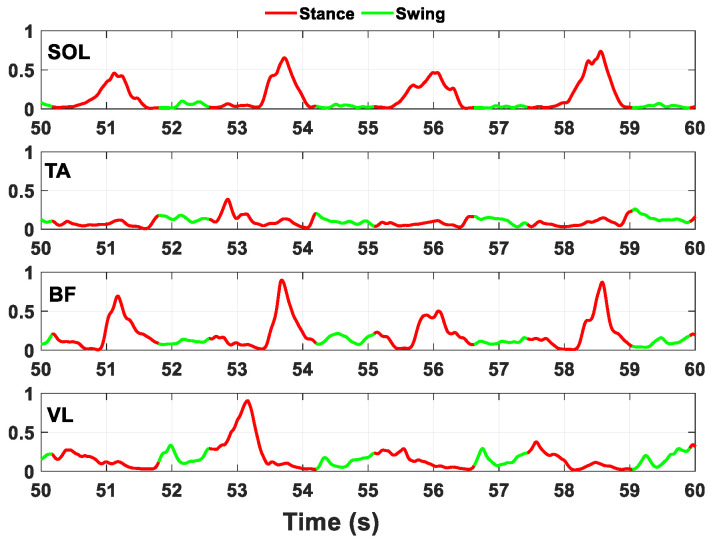
10 s example of the processed and labelled EMG signals of the four muscles (SOL, TA, BF, VL) during the stance (in red) and swing phases (in green) in the WO scenario.

In both datasets, all the obtained sequences were labelled, sample by sample, with one of the two classes indicating the stance and swing phases of the gait cycle (class 0 and class 1, respectively), in accordance with the previously described HA and KA data processing algorithm (see Figure 5).

Second experiment

Predicting that the pre-processing steps required to correctly down-sample the signal would increase the computational time required by the model prediction, we investigated using the 500 Hz-trained network for classifying non-down-sampled data (1500 Hz) while maintaining sequence length. 

Third experiment

Finally, to improve the model performance on non-down-sampled data, we partially retrained the model using a randomly selected subset of sequences drawn from the 1500 Hz WO dataset which accounted for 30% of the training data. This additional training was aimed at refining the model’s weights to adapt its classification ability to the novel representation of time and to the shorter time frame examined.

### 2.6. Deep Learning Neural Network

We employed a deep learning model implemented in Python 3.9 using the Keras API. The model is based on the bidirectional long short-term memory (BiLSTM) layer, a specialized recurrent neural network (RNN). Based on the high accuracies reported in the literature concerning LSTM-based architectures in classifying gait phases using EMG signals [48,49,50], we chose to explore its performance on our EMG data, still using a bidirectional model to exploit temporal dependencies in both forward and backward directions, which is crucial for analyzing highly stochastic time-series data like EMG signals.

The model’s core layer, a bidirectional LSTM, is characterized by 200 hidden units and encompasses 324,800 parameters. To counter overfitting, a corresponding 60% dropout layer was introduced.

Such a layer is followed by three additional bidirectional LSTM layers, each comprising 100 hidden units, interspersed with 60% dropout layers. All LSTM layers were configured to output sequences, and the final LSTM output sequence was fed to a cascade of three time-distributed fully connected layers composed of 100, 50, and 25 hidden neurons, respectively, which were also interspersed with 60% dropout layers to counteract overfitting and enhance generalization capabilities. The model’s design ends with a time-distributed dense layer with a softmax activation function producing the final predictions for stance or swing. The proposed architecture presents a total of 1,233,677 trainable parameters.

A NVIDIA GeForce RTX 3090 GPU (Nvidia Corporation, Santa Clara, CA, USA) was used to train the BiLSTM model. 

## 3. Results

### 3.1. First Analysis: Testing on 500 Hz Data

In the first analysis, the BiLSTM model was trained on the 500 Hz WO dataset and evaluated using both the 500 Hz WO and WT test datasets. Its performance is summarized in Table 3, considering the overall accuracy and class-specific measures, including its specificity, recall, precision, and F-score, specifically for the stance and swing classes. On the WO test dataset, the model showed an accuracy of 92.43%, with a specificity of 92.48% and 92.41% for the stance and swing classes, respectively, a recall of 92.41% and 92.48%, a precision of 96.15% and 85.71%, and an F-score of 94.24% and 88.97%. Similarly, for the WT test dataset, the model achieved an accuracy of 91.16%, with a specificity of 87.39% and 93.04% for the stance and swing classes, respectively, a recall of 93.04% and 87.39%, a precision of 93.99% and 85.54%, and an F-score of 93.51% and 86.46%.

**Figure 6:**
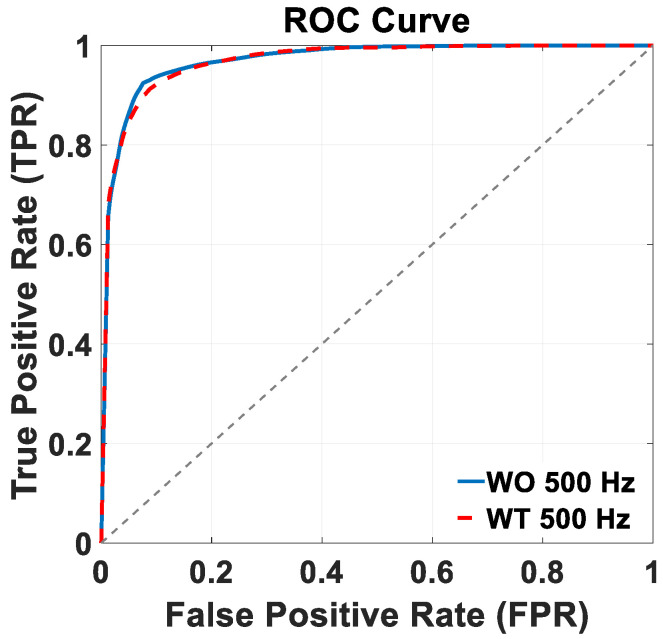
ROC curves referring to WO (blue line) and WT test datasets (dashed red line), both under-sampled at 500 Hz.

Figure 6 shows the ROC curves of the trained BiLSTM model version across the two distinct 500 Hz test datasets: WO (continuous blue lines), and WT (dashed red lines). Notably, in both the WO and WT scenarios, the trained model achieved an AUC value of 97%. Both ROC plots in Figure 6 and in the following section of this work were built considering the stance class as the “positive” class, so that the true positive rate (TPR) and false positive rate (FPR) need to be considered from that perspective. In terms of controlling the kinematics of the exoskeleton, though, errors regarding false positives and false negatives are likely to be just as detrimental; in other words, both misclassifications could expose the patient to the risk of falling. Based on these considerations, the best compromise is then represented by the top left corner of the curve. Additionally, as the goal of this model is to function as a practical choice for the online control of exoskeletons tailored to rehabilitation purposes, a real-time data preparation and gait phase classification simulation was implemented. The approximate time required for pre-processing and classifying a 1 s EMG signal from each muscle was found to be around 127 ms (58 ms for data preprocessing and 69 ms for classification).

### 3.2. Second Analysis: Testing on 1500 Hz Data

The results of the second analysis in which the same BiLSTM model, trained on the 500 Hz WO dataset, was tested on the two 1500 Hz WO and WT datasets are summarized in Table 4.

On the 1500 Hz WO dataset, the model showed an accuracy of 83.91%, with a specificity of 83.15% and 84.30% for the stance and swing classes, respectively, a recall of 84.30% and 83.15%, a precision of 90.98% and 72.44%, and an F-score 85.51% for the stance class and 77.43% for the swing class.

On the 1500 Hz WT dataset, the model achieved an overall accuracy of 86.21%, a specificity of 82.33% and 88.04% for the stance and swing classes, respectively, a recall of 88.04% and 82.33%, a precision of 91.35% and 76.46%, and an F-score of 89.66% for the stance class and 79.29% for the swing class. 

**Figure 7:**
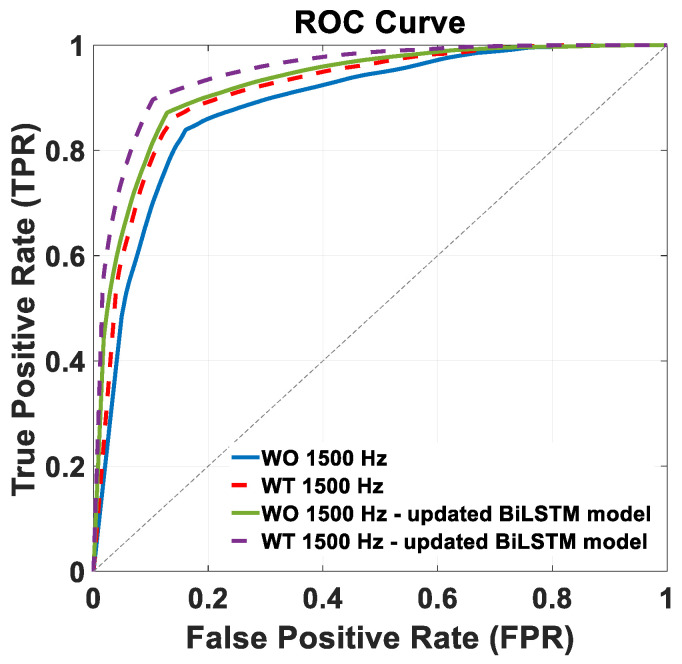
Comparison of ROC curves depicting the performance of the BiLSTM model on the 1500 Hz WO and WT test datasets. The performance of the original BiLSTM model is represented by the blue line (WO) and dashed red line (WT), while the retrained BiLSTM model is illustrated by the green line (WO) and dashed violet line (WT).

Figure 7 shows the ROC curves of the trained BiLSTM model version across the two distinct 1500 Hz test datasets: WO (continuous blue lines) and WT (dashed red lines). The AUC achieved in this condition were 89% and 91%, for the WO and WT datasets, respectively, with the corresponding ROC curves shown in Figure 7.

In this analysis, not requiring the down-sampling of the test data, the online simulation of the data pre-processing and classification was achieved in around 116 ms (47 ms for data pre-processing and 69 ms for classification), which is around 11 ms faster than the classification of the down-sampled data in the first analysis.

### 3.3. Third Analysis: Partial Retraining 1500 Hz

Table 5 details the performance metrics for the third analysis, in which the 500 Hz-trained BiLSTM model was retrained on 30% of the 1500 Hz WO training data and then evaluated on the two 1500 Hz test datasets.

On the WO dataset, the model achieved an accuracy of 87.17%, with specificity values for the stance and swing classes of 79.75% and 90.85%, respectively. The recall values for the stance and swing classes were 90.85% and 79.75%, and the precision values were 90.04% for the stance class and 81.22% for the swing class. The F-score values for the stance class and swing class were 90.44% and 80.48%, respectively.

Similarly, for the WT dataset, the model attained an accuracy of 89.64%, with specificity values for the stance and swing classes of 81.73% and 93.39%, respectively. The recall values were 93.39% and 81.73%, the precision values were 91.55% for the stance class and 85.36% for the swing class. The F-score values were 92.46 and 83.51% for the stance and swing classes, respectively.

Figure 7 shows the ROC curves of the retrained BiLSTM model version across the two distinct 1500 Hz test datasets: WO (continuous green line) and WT (purple dashed lines). The corresponding AUC values were 93% and 95%, respectively.

For the latter solution, we conducted a more detailed analysis by considering the EMG data of each of the three subjects of the test dataset. Each subject had seven acquisitions each for the WO and WT tasks. The quality of the EMG signal, upon a naked-eye examination, was dissimilar among the muscles and the subjects between the WO and WT data. 

**Figure 8:**
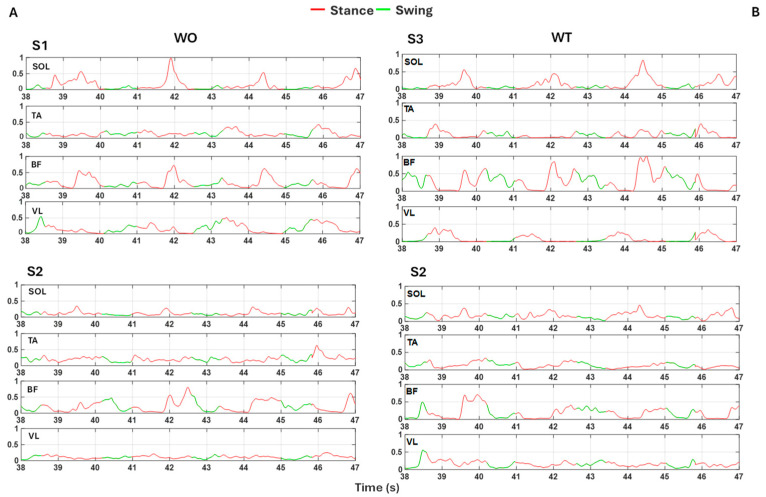
Examples of the best and the worst input signals to the BiLSTM model from the WO test set are shown in S1 and S2 (**A**) and examples from the WT test set are shown in S3 and S2 (**B**). Signals represent normalized muscular activation of the soleus (SOL), tibialis (TA), biceps femoris (BF), and vastus lateralis (VL) during four gait cycles. Red traces indicate time intervals labeled as belonging to the stance phase while green traces indicate those labeled as swing phases. The difference in signal quality is evident, especially in the SOL, TA, and VL signals of S2 in both scenarios (WO and WT), where identifying the steps and distinguishing the stance and swing phases is challenging even visually.

To provide a comprehensive metric that summarizes the performance of the trained network based on the varying quality of the input data, the retrained BiLSTM model was tested separately with each subject’s acquisition. Finally, for each test subject, a mean accuracy was computed for WO and WT, separately. The results of the mean accuracy are shown in Table 6, respectively, for each subject. The quality of the recorded data influences the model accuracy: for both WO and WT, S1 and S3 present a good-quality signal and obtain a higher mean accuracy value compared to S2, whose EMG input signals are of lower quality (Figure 8). The accuracies are generally higher with the WT recordings from the same subjects, although they were recorded on a different day from the WO ones and, hence, after a new positioning of the EMG electrodes. Despite such signal quality variability, the performance of the model is only minorly affected, proving the robustness of the proposed classifier.

## 4. Discussion

This paper outlines a methodology employing a deep learning model fed with EMG signals from four muscles of the dominant leg to discern the stance and swing phases of the gait cycle. The model learns with supervised training to distinguish the two gait phases with high accuracy and relatively swift processing times, approaching real-time capability. The intention is for this approach to be a viable choice in controlling exoskeletons designed for rehabilitation purposes. For the training and testing of the deep learning model, we chose to use EMG signals acquired on healthy subjects during walking with an exoskeleton for three main reasons. First, exoskeleton walking represents the hypothesized model-use scenario in which online signals are captured for controlling the torques to be generated by the device motors. Second, previous studies have demonstrated how robotic assistive devices alter muscle activation patterns during gait and reduce gait velocity, which in turn modifies the EMG signals [61,66,67,68,69]. Third, due to our muscle–skeletal system dynamics, muscular activations lead limb kinematics during gait by over 120 ms; [40,41] therefore, using EMG signals provides an opportunity to predict the torques needed to assist the patient’s gait in a timely manner.

The EMG data from four leg muscles of the dominant leg were collected on 26 normal subjects walking with an exoskeleton on both overground (WO) and treadmill surfaces (WT). In the first analysis, the data underwent pre-processing and down-sampling (from 1500 Hz to 500 Hz) to facilitate the model learning task by reducing the number of samples needed to analyze a sufficient time frame for disambiguating EMG signals. These were then segmented into 500-sample sequences, each with a 50% overlap, which represented the input to train the model.

When tested on the 500 Hz WO test dataset, the model showed an overall accuracy of 92.43%. Examining class-specific metrics provided a detailed understanding of the model’s performance (Table 1 of the Results section). In the WO dataset, the model demonstrated strong performance for both the stance, accurately identifying instances while minimizing errors across various metrics, and the swing classes, despite a slight imbalance in the class distribution within the augmented training dataset. This imbalance may cause the BiLSTM model to exhibit a prediction bias toward the stance class, as it is more prevalent in the training dataset.

Comparatively, on the 500 Hz WT test set, the model maintained strong performances, albeit slightly reduced, with an accuracy of 91.16%. This suggests that the model learned classification criteria that are valid in diverse scenarios, resulting in its ability to generalize well to a different walking condition task, one that the model had not seen in the training phase. Inspection of the class-specific metrics for the stance and swing classes in the WT test confirmed the model’s expertise in correctly identifying gait phases, with scores ranging from approximately 86% to 94% (Table 3 of the Section 3).

In a direct comparison between the WO and WT datasets, while there were minor variations in the specificity, recall, precision, and F-score metrics, the BiLSTM model showed robust adaptability and consistent performance across different walking conditions. The model’s discrimination capabilities for WO and WT scenarios achieved a remarkable AUC of 97%, with processing times for classification at around 127 ms. Despite the controlled setting provided by treadmill walking, the model’s capacity to generalize from overground training data implies its potential effectiveness in real-world situations as a valuable tool for torque control in exoskeleton-assisted rehabilitation training.

To assess the model’s adaptability to diverse data and examine the possibility of using it to classify signals requiring fewer pre-processing steps, the pretrained BiLSTM was also tested, without additional training, on 1500 Hz signals, i.e., non-under-sampled EMG data. The evaluation involved segmenting test sequences from the 1500 Hz WO and WT datasets into 500-sample sequences. In this context, the model demonstrated a decrease in performance for both the WO and WT datasets compared to those sampled at 500 Hz. The accuracy dropped by approximately 9% for the WO test dataset (from 92.43% to 83.91%) and by around 5% for the WT test dataset (from 91.16% to 86.21%). This decrease in accuracy can, at least partially, be attributed to the 500-sample training sequences, which contained signals equivalent to 1 s of acquisition when sampled at 500 Hz, and a duration of only 0.33 s when drawn from 1500 Hz-sampled data. This discrepancy in sequence duration during training and testing phases may lead to a reduced model performance as the model may struggle to generalize effectively across varying temporal resolutions. It is noteworthy that the WT test dataset exhibited a comparatively smaller decrease in accuracy, as reflected in the other metrics shown in Table 4 of the Results section.

This observation underscores the significance of the treadmill as a training environment when using an exoskeleton, as it provides a controlled condition that mitigates the inherent variability of the task compared to WO [70,71,72]. On the other hand, the simulation of online data pre-processing and classification gained 11 ms compared to the previously described 500 Hz setting, confirming that such an approach may be of interest for the intended use scenario if better prediction performances may be achieved. 

To improve the BiLSTM model’s performance on 1500 Hz data, we used 30% of the available 1500 Hz WO data for incremental model training. Such further learning of the model led to improved accuracies compared to the previous results: for the WO test dataset, the accuracy increased from 83.91% to 87.17%, and for the WT test dataset, it rose from 86.21% to 89.64%. These results confirm the effectiveness of the iterative training approach in enhancing the model’s performance, in line with the finding that incorporating additional training on specific subsets of the dataset, especially subsets with a higher temporal resolution, has proven beneficial in refining model accuracy [73].

The comparative analysis of the performance metrics presented in Table 4 and Table 5 of the Results section reveals substantial improvements in the retrained BiLSTM model, with increased specificity, recall, precision, and F-score values for both the stance and swing classes on the WO and WT datasets. The ROC curve analysis in Figure 7 of the Results section further supports these findings, indicating improved discriminative ability in the retrained BiLSTM model, with higher AUC values of 93% (WO) and 95% (WT) compared to the original model’s AUC values of 89% and 91%. Overall, these results suggest that further training significantly enhanced the model’s performance and discriminatory power across both test datasets.

In this scenario, we examined the accuracies obtained from individual acquisitions for each of the three test subjects, revealing how the performance of the model is only minorly affected by the quality of the EMG input data. This result is especially relevant considering this study’s goal of using the model for online exoskeleton control in patients with gait disorders, as they are most likely to present with suboptimal EMG signals. A potential future development would be to investigate and identify an indicator of the input signal quality that could predict the model’s performance.

Finally, when comparing our accuracies with those reported in the literature for gait phase detection based on EMG signals (97.89% [48], 95% [49], and 93.15% [50]), we found that our results are comparable, although slightly lower. On the other hand, comparison across the different studies is only possible to a limited extent, as the studies differ in terms of the number and selection of acquired muscles, signal processing, and dataset construction methodologies. In particular, [49,50] use a higher number of muscles, [49] uses time, frequency, and time-frequency domain features, and [48,50] use EMG time series but do not use a cross-subject design for training and testing, which has significant importance in terms of assessing expected generalization abilities.

As a more general consideration, while achieving high accuracy is important, our focus also includes the real-time classification of gait phases and the model’s ability to generalize both to new subjects and to different walking conditions. These represent a set of relevant objectives in assessing the applicability of the proposed solution that has not been addressed by other studies.

## 5. Conclusions & Feature Perspectives

In conclusion, this study presents a robust methodology based on a deep learning model fed with EMG signals to identify gait phases during exoskeleton-assisted walking, considering its application in real-time scenarios that are relevant to rehabilitation. The model, based on a BiLSTM, demonstrated promising accuracy (ranging from 83.91% to 92.43%) in distinguishing gait phases (stance and swing), and performs such computations in around 110 ms, making it a viable option for partial assistance exoskeleton control in rehabilitation.

Compared to the studies available in the literature, the one presented here introduces several novelties, including a new acquisition protocol, a different approach to gait data segmentation for building the dataset based on inertial signals, and a different goal of the classification, considering its generalization abilities from WO to WT scenarios and tailoring it to the control of the torques produced by the exoskeleton. These factors make the comparison of our results with those previously published not viable. However, it is essential to also acknowledge the limitations of this work. Labeling EMG data based on the kinematics of hip and knee joint angles introduces a delay compared to actual muscle activation, potentially leading to errors during model training, which we have not fully investigated. 

A basic approach could involve adjusting the labelling using the value reported in the literature (120 ms) [40], yet muscle activation timing can vary significantly between subjects, particularly in the presence of pathologies, such as multiple sclerosis or Parkinson’s disease. An ideal labelling approach would then have to be subject-specific, based on identifying and using each subject’s EMG and motion data, anticipating muscle activations relative to the kinematics, and defining an average value to adjust the labelling accordingly. However, determining this value is challenging due both to the non-stationary and stochastic nature of EMG signals, and to the complexity of designing an efficient experimental protocol that allows such measurement. 

A further limitation of this study is that the model has not been tested on gait data acquired on patients with lower limb impairments. Future work will include testing the model on patients with gait disorders.

Also, the filters used in pre-processing the EMG signals may not be the optimal choice for real-time processing, and that could pose challenges for online exoskeleton control applications. Furthermore, a cross-validation study replicating the selection of 23 subjects for training and 3 subjects for testing should have been carried out over the dataset to ensure the robustness of the model. Future work should focus on developing a larger database of subjects and including EMG data from both legs to enhance the model’s performance and applicability. An additional improvement could be the investigation of an index capturing the input EMG data quality to predict the model’s classification performance.

On the other hand, our findings underscore the importance of utilizing EMG signals acquired during exoskeleton-assisted walking, as they align with the altered gait dynamics induced by the device. The model testing encompassed both overground and treadmill walking conditions, proving its adaptability to the different scenarios. Notably, the model’s performance was also validated in a controlled treadmill setting, a common scenario in rehabilitation training, further highlighting the model’s potential real-world applicability.

While the best model performance was observed with EMG data sampled at 500 Hz (92.43% accuracy), its adaptability to varying conditions suggests versatility. The retrained model’s performance on signals sampled at 1500 Hz, achieving 89.64% accuracy, suggests potential for online applications, making it a promising tool for scenarios requiring dynamic motor task classification. 

Future investigations could further explore its applicability in rehabilitation robotics. Specifically, efforts could focus on two main goals. First, although the stance and swing phases are a crucial discrimination within the gait cycle, the ideal scenario for exoskeleton control would involve identifying sub-phases within the two main ones, allowing for finer control and adaptation during walking tasks. Second, future work should focus on identifying an optimal compromise between the input sequence duration, overlap, and processing times and the frequency of predictions provided to the exoskeleton motor control algorithm.

## Figures and Tables

**Table 1:** Inclusion and exclusion criteria.

Inclusion Criteria	Exclusion Criteria
Age between 18 and 35 years, broadly defined as adulthood. Weight below 95 kg. Ability to exercise safely (checked via the Health/Fitness Facility Pre-Participation Screening Questionnaire) [54].	Females currently pregnant. Currently seeking treatment. Any systemic, rheumatic or neuro-musculoskeletal disorders. Current use of anti-anxiety medications, muscle relaxants or tranquillizers. Relevant history of back or lower limb pain over the last three years.

**Table 2:** Exoskeleton’s walking parameters for this study.

	Parameter	Selected Value
Walking overground & Walking on treadmill	step length (cm)	40
	step height (cm)	15
	step duration (s)	1.2
	pause between the steps (s)	0

**Table 3:** Performance metrics of the BiLSTM model trained on 500 Hz WO dataset and tested on 500 Hz WO and WT test datasets. Performance indicators include overall accuracy and class-specific measures—specificity, recall, precision, and F-score—for the stance and swing classes.

Test Dataset	Sampling Rate (Hz)	Accuracy (%)	Specificity (%)		Recall (%)		Precision (%)		F-Score (%)	
		Overall	Stance	Swing	Stance	Swing	Stance	Swing	Stance	Swing
WO	500	92.43	92.48	92.41	92.41	92.48	96.15	85.71	94.24	88.97
WT	500	91.16	87.39	93.04	93.04	87.39	93.99	85.54	93.51	86.46

**Table 4:** Performance metrics of the BiLSTM model trained on 500 Hz WO dataset and tested on 1500 Hz WO and WT test datasets. Performance indicators include overall accuracy and class-specific measures—specificity, recall, precision, and F-score—for the stance and swing classes.

Test Dataset	Sampling Rate (Hz)	Accuracy (%)	Specificity (%)		Recall (%)		Precision (%)		F-Score (%)	
		Overall	Stance	Swing	Stance	Swing	Stance	Swing	Stance	Swing
WO	1500	83.91	83.15	84.30	84.30	83.15	90.98	72.44	85.51	77.43
WT	1500	86.21	82.33	88.04	88.04	82.33	91.35	76.46	89.66	79.29

**Table 5:** Performance metrics of the BiLSTM model retrained on 1500 Hz WO dataset and tested on 1500 Hz WO and WT test datasets. Performance indicators include overall accuracy and class-specific measures—specificity, recall, precision, and F-score—for the stance and swing classes.

Test Dataset	Sampling Rate (Hz)	Accuracy (%)	Specificity (%)		Recall (%)		Precision (%)		F-Score (%)	
		Overall	Stance	Swing	Stance	Swing	Stance	Swing	Stance	Swing
WO	1500	87.17	79.75	90.85	90.85	79.75	90.04	81.22	90.44	80.48
WT	1500	89.64	81.73	93.39	93.39	81.73	91.55	85.36	92.46	83.51

**Table 6:** Performance Evaluation of retrained BiLSTM model on test subjects’ 1500 Hz data acquisitions. The table presents the mean accuracy values obtained for each test subject, derived from separate analyses conducted for the WO and WT tasks.

Test Dataset	Sampling Rate (Hz)	Subject	Mean Accuracy Overall Acquisitions (%)
WO	1500	S1	92.96
		S2	86.33
		S3	88.97
WT	1500	S1	91.50
		S2	89.51
		S3	93.57

## Data Availability

The data will be made available to interested scientists upon request.
